# I deserve more A’s: A report on the development of a measure of academic entitlement

**DOI:** 10.1371/journal.pone.0239721

**Published:** 2020-09-30

**Authors:** Dennis L. Jackson, Marc P. Frey, Chelsea McLellan, Carolyn M. Rauti, Paige B. Lamborn, Jill A. Singleton-Jackson

**Affiliations:** Department of Psychology, University of Windsor, Windsor, Ontario, Canada; Aalborg University, DENMARK

## Abstract

This paper reports the results of a multi-stage effort to develop a measure of Academic Entitlement. An empirical/rational approach was taken to develop items and reduce the item set for a final version of the Academic Entitlement Scale (AES). The measure includes seven dimensions: Accommodation, Reward for Effort, Responsibility Avoidance, Grade Haggling, Customer Orientation, Customer Service Expectations, and General Academic Entitlement. Fit, using Confirmatory Factor Analysis, for the seven-factor correlated model and a bifactor model including General AE and the six specific factors, was good. The full measure is reported along with descriptive statistics for the scale and preliminary validation evidence.

## Introduction

Academic entitlement (AE) is a growing concern for university professors, staff, and administration. It is defined as the tendency for students to expect unearned academic success, undeserved academic services, and/or unrealistic academic accommodations [1, 2]. Although conceptualizations of the construct vary, expectations for reward absent from achievement, responsibility avoidance, and a consumerism approach to education appear to be consistent components of AE. Unreasonable expectations associated with excessive entitlement can lead to maladaptive behaviors, such as decreased work performance, productivity, and personal responsibility [3] and has the potential to negatively alter the university environment [4].

Defining AE and exploring correlates of AE have been the focus of research in the field of educational psychology for some time now, while measurement has received considerably less attention. Researchers have used a variety of strategies and tools to measure AE, which makes it difficult to compare findings from different studies. In this paper, we will review the existing AE literature; present a new multidimensional measure of AE, the Academic Entitlement Scale (AES); and discuss the validity and reliability of this measure.

Much of the existing AE research has focused on personality and individual differences or on students’ academic attitudes. Chowning and Campbell [1] for instance, found that AE is negatively correlated with agreeableness and conscientiousness, and McLellan and Jackson [5] found that AE is positively correlated with extroversion. Researchers have also suggested that AE is positively correlated with external locus of control and negatively correlated with self-efficacy and self-esteem [1, 6–10]. Academic entitlement is positively related to, but distinct from, narcissism and general psychological entitlement [1, 9, 11, 12]. Several researchers report that men tend to score higher on self-reported measures of AE compared to women [1, 7, 12, 13].

Exploring the relationship between AE and academic attitudes has revealed that AE is related to performance goal orientation [8, 11, 14] and grade orientation [15]. Past research has suggested that AE is related to negative academic outcomes such as higher levels of academic stress [16] and frustration [3] and it has shown to predict counterproductive research participant behavior, such as unexcused absence and careless survey responding [17].

There are mixed findings regarding whether AE is related to grade point average (GPA). Studies thus far have relied on self-reported GPA. While some studies report a negative relationship between AE and GPA [8, 13] others report a positive relationship [6] or no relationship at all [9]. Such contradictory findings could be due to differing definitions of AE as operationalized in the measures being used.

### Measurement of academic entitlement

Several measures of AE have been identified in our review of the literature. While some of these measures were well validated and are often used in research, others were used only by the developing author. Examples of well-developed, frequently used, validated measures include Chowning and Campbell [1] and Kopp and colleagues [10]. All of the AE measures reviewed contain items intended to assess students’ sense of entitlement to reward absent from achievement, avoidance of responsibility, and in most but not all cases, a general underlying expression of a consumer-oriented mindset or attitude, which denotes that entitlement can manifest itself through students seeing themselves as consumers rather than students [e.g., 2]. With this view, students may see themselves as being deserving of special treatment because they are paying for their education.

Although the first known measure of AE appeared in a dissertation [6], Greenberger et al. [9] presented the first published AE measure, a 15-item single-dimension scale. This measure captured the authors’ conceptualization of AE as expectation of reward for modest effort and a demanding attitude towards professors. Example items from this scale include: “If I have explained to my professor that I am trying hard, I think he/she should give me some consideration with respect to my course grade,” and “I feel I have been poorly treated if a professor cancels an appointment with me on the same day as we were supposed to meet.” Unfortunately, the structure of the scale has not been validated in any published work and researchers have questioned whether AE can be represented by a single dimension [8, 10, 14].

Chowning and Campbell [1] published a 15-item two-dimension measure of AE. Their measure appears to be the most popular choice to date. The two dimensions captured by this measure are Entitled Expectation and Externalized Responsibility. Across four studies, these authors presented evidence of scale validity and reliability and discussed the scale development process. The Externalized Responsibility subscale reflects perceptions of student and professor responsibilities in the learning process and includes items such as “It is unnecessary for me to participate in class when the professor is paid for teaching, not for asking questions,” and “If I do poorly in a course and I could not make my professor’s office hours, the fault lies with my professor.” The Entitled Expectations subscale reflects student expectations regarding policies and grading, for example, “My professors are obligated to help me prepare for exams” and “Professors must be entertaining to be good.”

A strength of Chowning and Campbell’s measure is its ability to predict behavior. In one study, AE predicted negative evaluations of a researcher; this is one of the only experimental studies to assess behavior associated with a measure of AE. Those with high scores on the Externalized Responsibility subscale were more likely to rate the researcher negatively regardless of receiving negative or neutral feedback. However, drawbacks of this measure are that the Entitled Expectations subscale consistently demonstrates moderate reliability (e.g., Cronbach’s α = .63) [1] and the measure contains double barreled items (i.e., “If I do poorly in a course and I could not make my professor’s office hours, the fault lies with my professor”) [13].

A newer measure of AE developed by Kopp et al. [10] has been used in more recent studies. These authors reviewed the existing literature on AE and identified five domains (originally outlined by Dubovsky; [18]). They then created items that mapped onto the five domains: “KR—Knowledge is a Right that should be delivered with a minimum of exertion and discomfort on the part of the ‘consumer’; OP—Others will Provide all of the education that is necessary; PL—Problems in Learning are due to the inadequacies of the teacher, the course, or the system, rather than to the student’s own shortcomings; SC—Students should have Control over class policies; and, DT—certain outcomes are Deserved because the student pays Tuition’ [18]. In terms of psychometric properties, these authors favoured a shortened measure over the five-dimensional measure presumably because they experienced convergence issues (in a confirmatory factor analysis; CFA) with the multidimensional measure. The shortened measure was considered more practical (i.e., easier to administer) and was psychometrically sound.

Some of the previous work has been well done and carefully validated. However, we felt that a different approach would be appropriate, one that was not purely theoretically driven (such as the Kopp et al. approach) and that was more expansive in terms of underlying dimensions. We used a rational/empirical approach, beginning with a large set of items identified as entitlement and applied theory, reason, and analytical techniques to determine a set of dimensions that we feel more fully encompass the AE construct.

### Rationale for another measure of academic entitlement

Most measures currently used have one or two subscales and, in our view, do not fully consider the role of the consumer model of AE. The Kopp et al. [10] measure was designed to have five subscales but the shortened unidimensional scale is used in research. Investigators conducting AE research should have access to a well-developed multidimensional measure and the ultimate utility of a measure such as the current one can be accessed empirically. In our validation efforts, we have observed interesting patterns of correlations between the subscales of our measure and other AE-related constructs. For instance, Responsibility Avoidance and Customer Service Expectations scales of the AES correlate more highly (*r*> .30) with a measure of attitudes toward academic dishonesty than Reward for Effort and Customer Orientation (*r* < .20). Also, the gender of the participant is more strongly related to Grade Haggling and Responsibility Avoidance (men score higher) and is relatively unrelated to Reward for Effort.

Further, in our approach, we chose to include some elements that might be considered outcomes of AE such as Grade Haggling, under the assumption that, while this may be a result of AE, it should also be viewed as a potential marker of AE. Our approach also included focus groups [2] consulting other measures, discussion with instructors, and first-hand experience. Diverse approaches to developing a measure will lead to a diversity of measures which is a healthy state for an emerging field. Researchers can use our measure to assess the antecedents and outcomes of AE, as well as identify students who may need AE intervention.

### Development of the Academic Entitlement Scale

The Academic Entitlement Scale (AES) reported in this paper is the culmination of several rounds of data collection. Published measures of AE were collected and items culled from those measures were assembled into a large question bank, which also included items written by the authors of this paper. Based on a review of the literature and consultation of associated literature (e.g., literature on entitlement and narcissism), a final set of items that were judged to come from the general construct of AE were piloted. What followed was four rounds of data collection and scale revision to arrive at the current instrument. Exploratory factor analysis was used early on to estimate the number of dimensions and to interpret those dimensions. Revisions in later rounds employed CFAs to test models for the AES. In the third round of data collection we arrived at a good fitting model but opted to reword, remove, and add items to improve the factor structure and remove the need to allow for some correlated residuals. Earlier versions of the scale included reverse-worded items which we did not use for creating scale scores, but which were useful for detecting inconsistent or careless responding. These reverse worded items were removed from the final version.

Previous versions of the AES have been shown to correlate in expected ways with other measures. For instance, dimensions of AES are correlated with psychological entitlement; academic self-efficacy; mastery learning orientation; mastery avoidance learning orientation; performance learning orientation; performance avoidance orientation; intrinsic motivation for knowledge and accomplishment, a motivation, academic self-esteem and attitudes toward academic dishonesty. Many of these findings were cross-validated over separate data collection efforts.

### Outcome expectations

A rationale and description of all models tested are listed in the S1 Appendix. First, we expected models containing all items, including reverse-worded items, to not fit as well as models excluding reverse-worded items. Previous work on our AE measure had demonstrated that the reverse worded items detracted from model fit [see also: 19]. We anticipated removing poor performing items based on analysis of the first sample (we randomly split our sample, see below). We wished to reduce the length of the questionnaire and had purposefully piloted more items than would be necessary for the final version. The Customer Orientation scale was weak in earlier versions of the instrument and additional items were written to strengthen it. Models were tested both with and without Customer Orientation and we had no *a priori* expectations about which would fit better once the new items were included. Finally, the General AE scale had previously been found to fit the data from the items defining it but we were not clear whether it would function as a correlated factor or a bifactor, so we tested both models. We suspected that our General AE factor measured trait entitlement rather than specific manifestations of AE because it was modeled off Campbell and colleagues Psychological Entitlement Scale, which is intended to measure trait-based entitlement [20]. A bifactor model, where items are specified to load on a general factor and on their specific factors [21], would allow us to partial out variance attributable to trait entitlement while a correlated factor model would not. Typically, researchers specify a bifactor model such that the bifactor is uncorrelated with other factors and does not contain its own unique manifest variables. In our case, the bifactor does contain its own defining variables and may be more properly referred to as an incomplete bifactor model [22]. This has an advantage of providing for an interpretation of the bifactor.

## Method

### Data cleaning

The total sample consisted of 1215 participants. Data cleaning procedures included voluntary withdraw, missing data analysis, time completion, response patterns, and response consistency [as outlined in 23]. We began by removing cases where participants had indicated they wished to have their data withdrawn (n = 10). We then removed cases with more than 10% missing data (n = 31) based on examining a histogram of missing data frequency among participants. This histogram revealed that after 10% missing data, the next case doubled to 20%, then to 40% and beyond. Overall, less than 3% of the sample was removed for excessive missing data and it is possible that those participants were qualitatively different in their responses, but it is also possible that they were attempting to gain participation bonus points with minimal effort. We then removed participants who took less than 5 seconds per question on average (n = 212). The completion time cut-off was established based on pilot testing, where participants were asked to complete the questionnaire as quickly as possible, reading each question. Next, we removed cases where there was no variability in responding in more than one of six blocks of 10 items (n = 7). Finally, we used six reverse-worded and positively worded pairs of items to construct a correlation coefficient to indicate response consistency and removed cases with inconsistent responses (n = 43). An example of paired items is “Professors should bend the rules for me” and “I do not deserve special treatment to help me perform better in class.” We used a cut-off (*r* = .55) corresponding to a one-tailed test *p* = .10. A total of 912 cases were retained for further analyses. The assumption of multivariate normality was not met. Due to the nature of the questions, distributions tended to be positively skewed.

### Participants

The participants in this study were 912 undergraduate students from a southwestern Canadian university. The majority of the sample self-identified as women (80.4%), with 19.1% identified as men, and 0.5% as non-binary. The average age of participants was 21 years old (*SD* = 4.35). The sample was primarily White or European-Canadian (69.6%), while 9.1% reported being of Middle Eastern descent, 6.6% Asian, and 4.7% African American/Canadian. Our sample consisted primarily of students from the Faculty of Arts, Humanities, and Social Sciences (41.2%), and was representative from all years of study (28.8% first year, 24.1% second year, 22.8% third year, and 24.0% fourth year or above). A small proportion of the sample were international students (2.9%). A comparison of demographic characteristics between the original and cleaned samples showed no differences in terms of age, ethnicity, program, year of study and international status. However, trivial differences were found for gender as data from males were slightly more likely to be removed during data cleaning.

The total sample was randomly split into two smaller samples consisting of 459 and 453 participants, respectively. There were no differences in demographic characteristics between these two samples. Participants were recruited from the psychology department’s participant pool where they received course credit for research participation. Informed consent was provided by participants electronically. This study was approved by the University of Windsor Research Ethics Board (REB).

### Measures

#### Academic Entitlement Scale (AES)

The AES (the focus of this paper) is a 30-item multidimensional measure of AE. This scale measures seven domains including General Academic Entitlement, Reward for Effort, Accommodation, Responsibility Avoidance, Customer Orientation, Customer Service Expectations, and Grade Haggling. Participants respond to items using a 7-point scale ranging from 1 (*strongly disagree*) to 7 (*strongly agree*). Cronbach’s alpha from previous versions of this questionnaire suggest good to excellent internal consistency with coefficients ranging from .75 to .95 [11].

#### Academic Entitlement Questionnaire (AEQ) [10]

The AEQ is an eight-item unidimensional measure of AE. Items on this scale include “It is the professor’s responsibility to make it easy for me to succeed,” and “Because I pay tuition, I deserve passing grades.” Participants respond to items using a 7-point scale ranging from 1 (*strongly disagree*) to 7 (*strongly agree*). The authors of this scale previously reported good reliability (ω = .81) and concurrent validity.

#### Pathological Narcissism Inventory (PNI) [24]

The PNI is a 52-item multidimensional measure that assesses aspects of narcissistic pathology. Due to an error when creating our online questionnaire only 50 items from this scale were included; “My self-esteem fluctuates a lot” and “When I get a glimpse of my needs, I feel anxious and ashamed” were unintentionally omitted. The PNI measures seven domains of narcissism: Exploitativeness, Grandiose Fantasy, Self-sacrificing Self-enhancement, Contingent Self-esteem, Hiding the Self, Devaluing, and Entitlement Rage. Items on this scale include “Everyone likes to hear my stories,” and “I often fantasize about being recognized for my accomplishments.” Participants respond to items using a 6-point scale ranging from 1 (*not at all like me*) to 6 (*very much like me*). This scale has previously demonstrated good reliability with Cronbach’s alphas ranging from .71 to .93 [25].

#### Legitimate Entitlement Questionnaire (LEQ) [26]

The LEQ measures students’ perceptions of their university campus, facilities, and faculty. A 13-item version was administered, but a reduced 7-item version was used for the subsequent analyses. Items on this scale include “The campus buildings and facilities are clean and well cared for,” and “Instructors in my classes are competent at their job.” Participants respond to items using a 7-point Likert scale ranging from 1 (*not at all true*) to 7 (*definitely true*). The LEQ has demonstrated good reliability (α = .81) in past research [26].

#### Demographics

The demographic questionnaire assessed participants’ gender, age, ethnicity, year of study, and program of study.

### Data analysis

Data were randomly split into two samples which were approximately equal in sample size and demographic characteristics. Three sets of CFAs were then conducted. The first sample (*N* = 459) was used to test a series of candidate models identified through analyses of data collected using a previous version of the AES, as well as to test post-hoc exploratory modifications (see S1 Appendix). The second sample (*N* = 453) was used to cross-validate the best fitting models from Sample 1. The two samples were then recombined and the final selected models were fit to the total sample to report the most stable parameter estimates.

CFAs were performed in R [27] using the Lavaan package, version .5–22 [28]. The maximum likelihood estimation procedure was used. Based on analyses with the previous version of the AES, we identified several candidate models that were tested using the first sample. Due to lack of conceptual clarity regarding the General AE factor, the best fitting model was tested twice: first with the general AE scale as a correlated factor, and second with the general AE scale as a general factor in the bifactor model. To evaluate model fit, we examined the chi-square statistic, Comparative Fit Index (CFI) [29], Tucker-Lewis Index (TLI) [30], Root Mean Square Error of Approximation (RMSEA) [31], Standardized Root Mean Square Residual (SRMR) [32], and Akaike Information Criterion (AIC) [33]. The fit indices reported are robust, calculated in accordance with the sample-based correction endorsed by Brosseau-Liard, Savalei, and Li [34] The following cut-off values were used to determine acceptable model fit: Chi-square p-value < .05, CFI and TLI >.95, RMSEA < .06, SRMR < .08 [35]. We also examined standardized residuals and factor loading magnitude to evaluate the suitability of various models.

## Results

### Confirmatory factor analyses

#### Sample 1

Table 1 provides the means and standard deviations of the total sample on the AES, PNI, LEQ and AEQ items. Table 2 contains the fit indices for all models tested in Samples 1 and 2, as well as the total sample. As expected, Model 1 (with all possible items, including reverse-coded items) had the poorest fit of all models tested. Though the fit improved by removing the reverse-coded items in Model 2, it was still deemed inadequate. Fit improved marginally after all subscales except Customer Orientation were reduced to include four items each (Model 3). The items were reduced based on the magnitude of factor loadings and paying attention to item content. In our previous work on this measure, the Customer Orientation factor in the AES showed poor reliability and low factor loadings. As such, two items intended to strengthen the Customer Orientation factor were added in Model 3. Based on the item loadings in Model 3, the Customer Orientation factor was reduced in Model 4, resulting in acceptable fit (CFI = .95, TLI = .94, RMSEA = .05 [.04 –.05], SRMR = .05, AIC = 34664).

**Table 1:** Means and standard deviations (total sample).

Scale Subscale							
	Summed Scores			Averaged Scores			
**AES**	*M*	*SD*	Min-Max	*M*	*SD*	Min-Max	α/**w**
General AE	15.71	7.01	6–42	2.61	1.16	1–7	.87/**.87**
Reward for Effort	12.22	5.33	4–28	3.05	1.33	1–7	.83/**.83**
Accommodation	7.58	3.52	4–28	1.89	.88	1–7	.75/**.77**
Responsibility Avoidance	6.65	3.23	4–28	1.66	.81	1–7	.68/**.70**
Customer Orientation	14.39	5.04	4–28	3.59	1.26	1–7	.70/**.71**
Customer Service	7.01	3.51	4–28	1.75	.88	1–7	.77/**.79**
Grade Haggling	7.21	3.87	4–28	1.80	.97	1–7	.82/**.83**
**PNI**							
Contingent Self Esteem				2.92	1.15	1–6	.93
Exploitative				3.04	.94	1–6	.77
Self-Sacrificing Self-Enhancement				3.77	.90	1–6	.77
Hiding the Self				3.97	.99	1–6	.74
Grandiose Fantasy				3.91	1.12	1–6	.87
Devaluing				2.72	1.02	1–6	.85
Entitlement Rage				2.94	.99	1–6	.86
**AEQ**	20.52	7.76	8–56				.81
**LEQ**	33.35	6.47	7–49				.81

**Table 2:** Fit indices of models tested in Sample 1, Sample 2, and the total sample.

Model	Scaled χ^2^	df	CFI	TLI	RMSEA [90% CI]	SRMR	AIC
**Sample 1 (*N* = 459)**							
Model 1	1913.19	930	.85	.84	.05 [.05, .06]	.06	66881
Model 2	1175.58	614	.90	.89	.05 [.05, .06]	.06	53336
Model 3	606.73	334	.93	.92	.05 [.04, .05]	.05	40001
Model 4	441.04	260	.95	.94	.05 [.04, .05]	.05	34664
Model 5	571.05	309	.93	.93	.05 [.04, .06]	.05	37168
Model 6	406.60	259	.96	.95	.04 [.03, .05]	.05	34148
Model 7	406.42	260	.96	.95	.04 [.03, .05]	.05	34146
Model 8	359.07	237	.96	.96	.04 [.03, .05]	.04	32720
Model 9	617.75	384	.95	.94	.04 [.04, .05]	.05	41283
Model 10	590.21	366	.95	.94	.04 [.04, .05]	.04	41280
**Sample 2 (*N* = 453)**							
Model 8	375.53	237	.95	.94	.04 [.04, .05]	.05	32941
Model 9	636.88	384	.94	.93	.05 [.04, .05]	.05	41438
Model 10	580.45	366	.95	.94	.04 [.04, .05]	.04	41391
**Total Sample (*N* = 912)**							
Model 8	492.21	237	.96	.95	.04 [.04, .05]	.04	65617
Model 9	877.49	384	.94	.94	.04 [.04, .05]	.04	82653
Model 10	810.20	366	.95	.94	.04 [.04, .05]	.04	82593

Two new items were added to the model, which had been written after analyses of the previous version of the AES; one on Reward for Effort and the other on Customer Service, which resulted in a decrement in fit (Model 5). However, fit improved after further reducing the subscales and allowing an item (“If I do poorly in a course, the fault lies with my professor”) to cross-load onto both Responsibility Avoidance and Accommodation, as suggested by the modification indices (Model 6). Subsequently, we trimmed the path of this item so that it only loaded on Accommodation (Model 7) and removed another item from Accommodation (“A professor should modify course requirements to help me achieve a better grade”) so that all factors had four items. This model (Model 8) had the best fit of any model tested in Sample 1 (CFI = .96, TLI = .96, RMSEA = .04 [.03 - .05], SRMR = .04, AIC = 32720), with all fit indices meeting or exceeding conventional cut-offs.

To investigate the influence of the General AE subscale, we added it as a correlated factor to the previous model solution (Model 9), which resulted in decremented, but still acceptable fit (factor loadings are shown in Table 3). However, we suspected that the General AE factor measured trait entitlement rather than specific manifestations of AE. To attempt to model this trait aspect, we tested a bifactor model (Model 10). The six unique General AE items and all the other items in the scale loaded onto an orthogonal general factor, and the non-general items also loaded onto their specific factors. This model showed adequate fit (factor loadings are shown in Table 4), and consistent with the trait interpretation of the General factor, attenuated the correlations among the specific AE latent variables.

**Table 3:** Factor loadings for the correlated 7-factor model (Model 9).

Subscale		Factor Loadings		Item
# Item		Standardized	Unstandardized	R^2^
**Reward for Effort**				
1	Even if I do not perform well, I should get a good grade if I worked hard.	.78	1.00^a^	.60
2	I should get an A for attending all lectures and completing all of the course material.	.79	1.05	.62
3	I should never fail an assignment I put effort into.	.69	1.02	.47
4	If I have completed most of the reading for a class, I deserve a good grade.	.74	0.89	.54
**Accommodation**				
5	Professors should bend the rules for me.	.75	1.00^a^	.56
6	My test date should be moved if I am not prepared.	.72	1.10	.51
7	I should not have to think too hard to learn the material for a class.	.60	1.05	.36
8	If I do poorly in a course, the fault lies with my professor.	.61	0.98	.38
**Responsibility Avoidance**				
9	I am not motivated to put effort into group work, because another group member will end up doing the work.	.55	1.00^a^	.30
10	For group assignments, it is acceptable to take a back seat and let others do most of the work.	.72	1.15	.53
11	It is acceptable to lie to a professor if it helps me to avoid failing an assignment.	.60	1.34	.36
12	In group assignments, I should receive the same grade as the other group members regardless of my level of effort.	.56	1.20	.32
**Customer Orientation**				
13	I deserve to have more input in how my classes are taught.	.61	1.00^a^	.37
14	I should be able to choose how my knowledge is tested.	.71	1.21	.51
15	I ought to be able to choose the courses required for my degree.	.56	1.05	.31
16	I deserve to be entertained by my professors’ lectures.	.57	0.96	.32
**Customer Service Expectation**				
17	A professor should be willing to meet with me at a time that works best for me, even if inconvenient for the professor.	.61	1.00^a^	.38
18	Professors should respond to e-mails within 30 minutes.	.62	1.00	.38
19	I should be able to call my professor at home if I need help.	.79	1.07	.62
20	I should have my instructor’s cell phone number to contact him or her if I need help.	.75	1.00	.56
**Grade Haggling**				
21	No tactic is too extreme when arguing for an improved grade.	.69	1.00^a^	.48
22	Professors just make grades up, so it is not a problem to argue for a higher grade.	.81	0.99	.66
23	I always deserve a higher grade than I am given, making it necessary to argue for extra points.	.85	1.07	.73
24	Students should complain to the Dean or higher level of authority to get the grade they want.	.61	0.82	.38
**General Academic Entitlement**				
25	Great academic success should just come to me.	.52	1.00^a^	.27
26	I am worthy of more praise from my professors.	.64	1.22	.41
27	If a professor were only allowed to give one “A” in a course, it should be given to me.	.68	1.42	.46
28	I honestly feel I am more deserving than other students.	.79	1.54	.62
29	I demand the best grades because I deserve them.	.84	1.83	.71
30	I deserve more A’s	.84	1.94	.71

^a^The variable loading was set to 1.0 to scale the latent factor.

**Table 4:** Factor loadings for the bifactor model (Model 10).

		Factor Loadings				
Subscale		Standardized		Unstandardized		Item R^2^
#	Item	Specific	Bifactor	Specific	Bifactor	
**Reward for Effort**						
1	Even if I do not perform well, I should get a good grade if I worked hard.	.56	.55	1.00^a^	1.19	.61
2	I should get an A for attending all lectures and completing all of the course material.	.57	.55	1.06	1.22	.62
3	I should never fail an assignment I put effort into.	.53	.45	1.10	1.12	.48
4	If I have completed most of the reading for a class, I deserve a good grade.	.45	.57	0.76	1.17	.53
**Accommodation**						
5	Professors should bend the rules for me.	.57	.50	1.00^a^	0.68	.57
6	My test date should be moved if I am not prepared.	.57	.44	1.17	0.70	.52
7	I should not have to think too hard to learn the material for a class.	.41	.43	0.95	0.77	.36
8	If I do poorly in a course, the fault lies with my professor.	.44	.42	0.93	0.68	.37
**Responsibility Avoidance**						
9	I am not motivated to put effort into group work, because another group member will end up doing the work.	.52	.21	1.00^a^	.30	.31
10	For group assignments, it is acceptable to take a back seat and let others do most of the work.	.66	.30	1.11	0.38	.53
11	It is acceptable to lie to a professor if it helps me to avoid failing an assignment.	.51	.30	1.21	0.53	.35
12	In group assignments, I should receive the same grade as the other group members regardless of my level of effort.	.52	.22	1.19	0.37	.32
**Customer Orientation**						
13	I deserve to have more input in how my classes are taught.	.49	.36	1.00^a^	0.82	.37
14	I should be able to choose how my knowledge is tested.	.64	.36	1.37	0.84	.54
15	I ought to be able to choose the courses required for my degree.	.45	.31	1.06	0.81	.30
16	I deserve to be entertained by my professors’ lectures.	.44	.34	0.93	0.77	.31
**Customer Service Expectation**						
17	A professor should be willing to meet with me at a time that works best for me, even if inconvenient for the professor.	.50	.34	1.00^a^	0.57	.36
18	Professors should respond to e-mails within 30 minutes.	.50	.34	0.99	0.57	.37
19	I should be able to call my professor at home if I need help.	.73	.32	1.23	0.46	.64
20	I should have my instructor’s cell phone number to contact him or her if I need help.	.73	.26	1.20	0.37	.60
**Grade Haggling**						
21	No tactic is too extreme when arguing for an improved grade.	.54	.42	1.00^a^	0.76	.47
22	Professors just make grades up, so it is not a problem to argue for a higher grade.	.71	.43	1.11	0.65	.69
23	I always deserve a higher grade than I am given, making it necessary to argue for extra points.	.62	.57	1.00	0.88	.71
24	Students should complain to the Dean or higher level of authority to get the grade they want.	.55	.31	0.94	0.51	.40
**General Academic Entitlement**						
25	Great academic success should just come to me.	-	.52	-	1.00^a^	.27
26	I am worthy of more praise from my professors.	-	.65	-	1.23	.42
27	If a professor were only allowed to give one “A” in a course, it should be given to me.	-	.68	-	1.42	.46
28	I honestly feel I am more deserving than other students.	-	.78	-	1.54	.61
29	I demand the best grades because I deserve them.	-	.84	-	1.82	.71
30	I deserve more A’s	-	.84	-	1.94	.71

^*a*^ The variable loading was set to 1.0 to scale the latent factor.

#### Sample 2

Based on the fit indices, Models 8, 9, and 10 were tested in Sample 2. The fit of Model 8 (CFI = .95, TLI = .94, RMSEA = .04 [.04 - .05], SRMR = .05, AIC = 32941) was comparable to that of Model 10 (CFI = .95, TLI = .94, RMSEA = .04 [.04 - .05], SRMR = .04 AIC = 41391). Model 9 had slightly worse fit than Models 8 and 10, though still acceptable fit (see Table 2).

#### Total sample

Models 8, 9, and 10 were also tested in the total sample. Model 8 had the best overall fit, which was consistent with both Sample 1 and Sample 2 (CFI = .96, TLI = .95, RMSEA = .04 [.04 - .05], SRMR = .04, AIC = 65617). Model 10 had slightly worse fit, followed by Model 9 (see Table 2).

Using General AE in the bifactor model reduced the relationships among the specific AE factors and reduced the variance of the specific AE factors. All six of the specific AE factors still had significant variance in the bifactor model. The average reduction in variance was 35% and it was highest for Reward for Effort (49%) and Accommodation (43%), and lowest for Responsibility Avoidance (11%). On average, including the bifactor reduced the specific AE factor covariances by 55%. The largest reduction in covariances was for Reward for Effort’s relationship with both Responsibility Avoidance (76%) and Grade Haggling (73%). The smallest reductions were observed for covariances involving Responsibility Avoidance.

### Correlation analyses

To examine the relationship between the AES factors and related constructs, subscale scores were computed by summing the items on each factor. We used only the six items designed to identify the General AE factor to create its scale score. Bivariate correlations between the AES scores and those from other measures are reported below (see Table 5).

**Table 5:** AES and PNI, AEQ, LEQ Inter-correlations.

	1	2	3	4	5	6	7
1. General AE	--						
2. Reward for Effort	.60^a^	--					
3. Accommodation	.56^a^	.57^a^	--				
4. Responsibility Avoidance	.35^a^	.31^a^	.57^a^	--			
5. Customer Orientation	.44^a^	.56^a^	.46^a^	.26^a^	--		
6. Customer Service	.40^a^	.40^a^	.59^a^	.54^a^	.40^a^	--	
7. Grade Haggling	.50^a^	.48^a^	.65^a^	.57^a^	.43^a^	.63^a^	--
CSE	.25^a^	.20^a^	.25^a^	.20^a^	.20^a^	.21^a^	.16^a^
EXP	.26^a^	.10^a^	.16^a^	.18^a^	.20^a^	.19^a^	.18^a^
SSSE	.25^a^	.23^a^	.12^a^	.08^b^	.19^a^	.11^a^	.09^a^
HS	.11^a^	.06	.08^b^	.05	.06	.05	.01
GF	.29^a^	.17^a^	.14^a^	.14^a^	.19^a^	.12^a^	.15^a^
DEV	.32^a^	.23^a^	.30^a^	.27^a^	.26^a^	.28^a^	.25^a^
ER	.41^a^	.28^a^	.32^a^	.26^a^	.27^a^	.30^a^	.28^a^
LEQ	-.06	-.13^a^	-.23^a^	-.20^a^	-.22^a^	-.14^a^	-.20^a^
AEQ	.45^a^	.60^a^	.63^a^	.41^a^	.57^a^	.54^a^	.57^a^

CSE = Contingent Self-Esteem, EXP = Exploitative, SSSE = Self-Sacrificing Self-Enhancement, HS = Hiding the Self, GF = Grandiose Fantasy, DEV = Devaluing, ER = Entitlement Rage, LEQ = Legitimate Entitlement Questionnaire, AEQ = Academic Entitlement Questionnaire.

^a^Correlation is significant at the .01 level.

^b^Correlation is significant at the .05 level.

#### Pathological narcissism

On average, the General AE factor was most highly correlated with the PNI subscales compared to the other AES subscales, which supports the interpretation of the General AE scale as trait-based AE. Consistent with this interpretation, the highest correlation among the General AE scale and PNI subscales was for PNI Entitlement Rage (*r* = .41). The specific AES subscales had the lowest correlations with the PNI Hiding the Self (*r* ranging between .01 and .11). In contrast, the specific AES subscales were most highly correlated with Entitlement Rage and Devaluing.

#### Legitimate entitlement

Factor correlations between the AES and AEQ scale [10] were computed by simultaneously modeling both measures in a CFA and examining the factor correlations. The General AE factor correlated with the AEQ (*r* = .45), and the specific subscales ranged from *r* = .41 (Responsibility Avoidance) to *r* = .63 (Accommodation). The AES subscales generally had low, negative correlations with the LEQ. These findings suggest that there was a slight tendency for students who score higher on dimensions of AE to feel that their university is not meeting their expectations regarding the things they are legitimately entitled to receive. Conversely, there was a slight tendency for students who score lower on AE to feel their school is doing a good job of providing things, such as adequate and accessible educational resources and competent instructors.

#### Age and gender differences

Bivariate correlations computed between age and AES subscales were all non-significant (*p-*values>.05), except for customer expectation which was negatively related to age (*r* = -.13). Additionally, ANOVA results suggest that AES subscale scores do not differ by year of study. In general, the AES subscale scores do not appear to be related to age or to year or study.

To assess gender differences, t-tests were conducted (see Table 6). Statistically significant gender differences were observed for four AES subscales (grade haggling, customer expectation, accommodation, and responsibility avoidance), in all cases men tended to report higher AES scores than women. To further evaluate these differences, we calculated an upper tail ratio effect size [see e.g., 36]. The subscales were recoded into dichotomous variables (1 = one standard deviation above to mean or higher, 0 = lower than one standard deviation above the mean). Odds were calculated to determine whether men were more likely to be at least one standard deviation above the mean (for all AES scales) compared to women. For all but two AES subscales men were more likely than women to score at least one standard deviation above the mean (i.e., to have higher AES scores). For instance, men are 1.52 times more likely than women to have scores one standard deviation or higher above the mean for Grade Haggling. Women were more likely to score at or above one standard deviation above the mean compared to men on the Reward for Effort and Customer Orientation AES scales. MANCOVA results revealed that gender differences on the AES subscales remain after partialling some PNI subscales (i.e., Hiding the Self, Devaluing, and Entitlement Rage; p-values < .01) but not others (i.e., Contingent Self-Esteem and Grandiose Fantasy; p-values >.05).

**Table 6:** Mean gender differences in study scales.

Subscales	Descriptive Statistics					t-test Results		
	Men		Women					
	n = 174		n = 733					
	M	SD	M	SD	*t*	*df*	*p*	*UTR*
General AE	2.73	1.21	2.59	1.16	1.52	904	.13	1.33
Grade Haggling	2.02	1.22	1.75	0.89	2.69	216.89^a^	< .05	1.52
Customer Expectation	1.95	0.99	1.71	0.84	2.81	235.66^a^	< .05	1.77
Accommodation	2.02	0.97	1.86	0.86	2.19	904	< .05	1.76
Responsibility Avoidance	1.94	1.03	1.60	0.73	4.18	214.55^a^	< .05	1.42
Reward for Effort	2.94	1.39	3.08	1.32	-1.00	904	.32	0.86
Customer Orientation	3.48	1.18	3.63	1.28	-1.37	904	.17	0.69
CSE	2.85	1.12	2.93	1.16	.88	905	.38	.74
EXP	3.46	1.00	2.94	.90	6.74	905	< .05	2.73
SSSE	3.78	.94	3.77	.90	.14	905	.89	1.04
HS	3.88	.94	3.99	1.00	1.26	905	.21	.73
GF	4.13	1.11	3.86	1.11	2.87	905	< .05	1.32
DEV	2.71	1.01	2.72	1.02	.19	905	.85	.99
ER	2.97	1.02	2.92	.99	.58	905	.56	1.18
LEQ	33.65	6.53	33.26	6.46	.71	905	.48	1.07
AEQ	21.08	8.14	20.39	7.67	1.06	905	.29	1.19

*UTR* = Upper Tail Ratio and demonstrates the likelihood that males in appear one standard deviation above the mean relative to females.

^a^Indicates corrections for unequal variances.

## Discussion

Defining AE and exploring its correlates has been the focus of research in the field of educational psychology, while measurement of AE has received considerably less attention. The purpose of the present study was to report on the development of a new measure, the AES, and provide evidence of construct validity (factor structure) and concurrent validity via the relationship between the AES and other measures including narcissism (PNI), legitimate entitlement (LEQ), and maladaptive AE (Kopp et al.’s AEQ).

In the present study, we identified seven domains that define the construct. As anticipated from earlier rounds of scale development [as discussed in 14] the results from the current study suggest that AE should be treated as a multidimensional construct. Further, the findings suggest that AE can be measured as a general trait that is related to but distinct from more specific manifestations of AE. The resulting 30-item AES demonstrated psychometrically sound properties and has important implications, as past research has linked AE to maladaptive behaviors and student outcomes.

### Domains of academic entitlement

The seven domains of the AES are General AE, Reward for Effort, Accommodation, Customer Orientation, Customer Service, Responsibility Avoidance, and Grade Haggling. These domains align with aspects of previous conceptualizations of the AE construct that were discussed in the introduction of this paper. For instance, students’ expectations of unearned academic success are inherent in our subscales measuring Reward for Effort, Responsibility Avoidance and Grade Haggling. Students’ expectations of unearned or undeserved academic services are inherent in our subscales measuring Customer Orientation and Customer Service. And finally, students’ expectations of unrealistic accommodations are captured in our subscale measuring Accommodation. We modeled our General AE subscale off work by Campbell and colleagues [1] so our conceptualization is consistent with theirs–academic entitlement is a stable and pervasive sense in that, compared to others, one is more deserving and is entitled to more in an academic setting. Results from the bifactor model and correlations with the PNI support the trait interpretation of the General AE factor. This finding suggests that some students may possess entitled attitudes and behaviors in academia due to entitlement that is likely a broader part of their personality.

### Which model?

**Fig 1:**
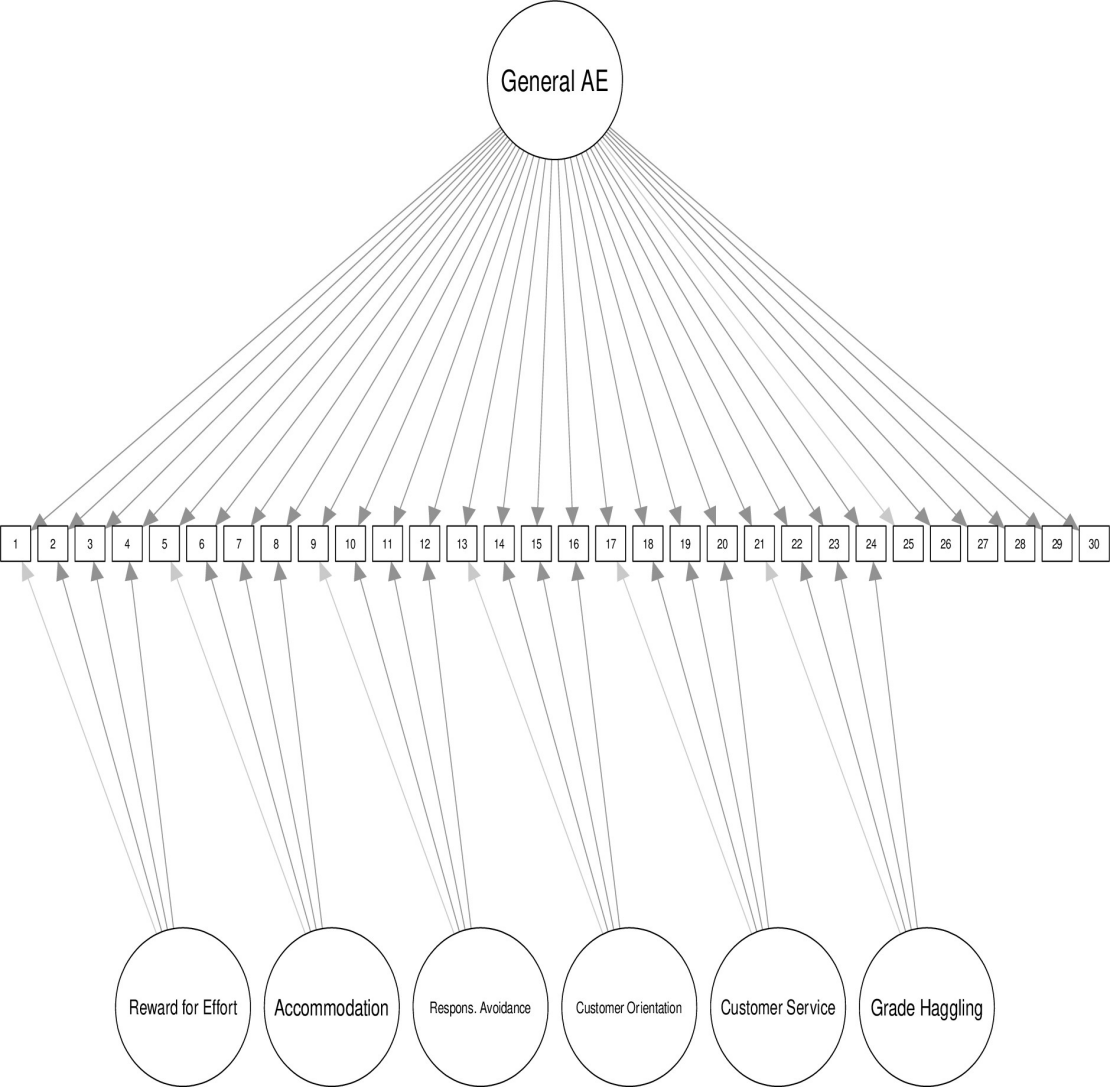
Model diagram of preferred model. Due to space constraints, factor correlations have been left off the model diagram.

Of the three final models, our preference is for Model 10 (Fig 1), the bifactor model including General AE. However, we feel that different research endeavors might result in a different choice of model. In SEM research, Model 10 has the advantage of partialing out trait variance from the specific AE constructs so that relationships among AE constructs and other variables of interest can be examined separately for trait AE and specific AE factors. For instance, one might want to know the relationship between locus of control and General AE, as well as Grade Haggling, but with the latter having variance attributable to General AE removed. In some research projects, either Model 8 or Model 9 is appropriate, depending upon whether the researcher wishes to use General AE in analyses. In this case, summed or mean scores for each construct could be used for analyses with other variables. Although researchers might choose Model 8, and choose to administer only the items for the six primary factors, we highly recommend using all items (Model 9) so that General AE scores are available for analyses and so that further research can shed light on the usefulness of General AE in research involving academic entitlement.

The bifactor model warrants further discussion. As would be expected, including General AE as a bifactor resulted in reductions in latent variable correlations and variances among the six specific latent AE variables. As noted above, and observable in Table 4, General AE overlaps more with Accommodation and Reward for Effort than it does with the other latent variables. The standardized loadings on these two latent variables are roughly equivalent to what they are on the General AE bifactor. The loadings of items on the remaining latent variables are appreciably higher than they are for the General AE bifactor. The measured variable R^2^ values changed very little by specifying General AE as a bifactor, meaning that this specification did not result in explaining new variance amongst the measured variables, but shifted some variance away from the specific factors to General AE. This implies that the model chosen for subsequent research could affect the interpretations made by researchers. In the bifactor model, the specific latent variables are more independent of each other and more independent of trait variance.

### Correlates of academic entitlement

Our findings indicated that, compared to specific AES subscales, the General AE factor was more highly correlated with traits of narcissism, supporting the notion that the General AE subscale measures more trait-based entitlement. The specific AES subscales had the lowest correlations with the Hiding the Self subscale of the PNI. This seems appropriate as AE generally involves the assertion of personal needs and desires rather than concealing these expectations from others. Further, the AES subscales were most highly correlated with the Entitlement Rage and Devaluing subscales of the PNI, constructs that are more consistent with the presentation of and constructs subsumed by AE.

The general AE and the six specific AES domains were highly correlated with Kopp et al. [10] AEQ. With respect to the six specific factors, the lowest correlation was for the Responsibility Avoidance Subscale and the highest was for Accommodation. These findings provide evidence for validity of the AES and the multidimensional nature of AE more generally. The AES subscales generally had low, negative correlations with the LEQ. As expected, these findings suggest that students who score higher on aspects of AE tend to feel that their university is not adequately meeting their expectations regarding the things they are legitimately entitled to receive, such as clean and well cared for classrooms, fair evaluations by instructors, competent instruction, and affordable food services. However, the relatively low magnitude of these correlations indicate that AE is largely independent of legitimate entitlement. This finding suggests that AE is not the opposite of legitimate entitlement and may suggest that university environments that exceed or fail to meet student expectations regarding legitimate entitlement will have at best a small effect on AE.

## Limitations and recommendations for future research

Future research should focus on replicating the current study using students from a variety of educational institutions. The AES was developed primarily at one institution, with some data gathered from a second. Also, invariance testing across populations, such as by gender and year of study would indicate the appropriateness of mean-level comparisons of AE. The notion of legitimate entitlement is a fairly novel portion of this study and more work needs to be done in this area. The low correlations either suggest relative independence of AE and legitimate entitlement, or that a search for moderators should be undertaken. For instance, motivation toward seeking an education could interact with legitimate entitlement to produce higher or lower AE.

Finally, we recommend that future research employ an experimental design to assess the relationship between AE and student outcomes. AE research using an experimental approach is currently very limited, and thus, causal links between AE and student outcomes have not adequately been addressed. For instance, past researchers have concluded that AE does not pay off in terms of higher grades [e.g., 9] however, these conclusions have been drawn from correlational research that relied on self-reported GPA which limits accuracy and generalizability of these findings. We also recommend that future research examines AE using a longitudinal approach to assess changes in students’ entitled attitudes and behaviors over time, and to assess longer-term educational outcomes such as degree completion.

## Supporting information

S1 Appendix Description of models. (PDF)
