# Supplementary material for: I deserve more A’s: A report on the development of a measure of academic entitlement
Source: PLoS One. 2020 Sep 30;15(9):e0239721. doi: 10.1371/journal.pone.0239721 (PMC7526903; doi:10.1371/journal.pone.0239721)
Supplement: S1 Appendix — (PDF) [file pone.0239721.s001.pdf]

# S1 Appendix: Description of Models

Four rounds of data collection and scale revision were used to arrive at the final AES. All models reported here were analyzed on data collected in the final round, although previous scale versions and analyses were used to inform model decision-making. Models 1 and 2 were focused on re-establishing a correlated six-factor model that had been supported in analyses of previous versions of the AES. One of the factors, Customer Orientation, was shown to be weak in previous versions of the AES; as such, models 3 and 4 reflect our attempts to strengthen this factor with new items and reduce the number of items on each factor. Models 5 through 8 focused on fine-tuning the model by adding items that were new in this final version of the AES, examining cross-loading items, and re-trimming the models so that each factor had four items. Finally, Models 9 and 10 were aimed at examining the General AES subscale, first as a correlated factor and then in an incomplete bi-factor model.

*Model 1: Full model*. This model represents the best solution from the previous version of the AES, but includes reverse-coded items, which were found not to fit well in previous versions. Between 7 and 8 items were specified to load onto six correlated factors. Four new items were added to the questionnaire for the final round of data collection. However, to start with a model that is consistent with the of the questionnaire, we did not include these new items until otherwise noted below.

*Model 2: Full model, without reverse-coded item*. We excluded all reverse-coded items from the previous model, leaving between 6 and 7 items on each factor.

*Model 3: Reduced model, with full Customer Orientation scale*. The following subscales were reduced to contain between four and five items each: Reward for Effort, Accommodation, Responsibility Avoidance, Customer Service, and Grade Haggling. The items were removed based on item loadings in analyses conducted with the previous version of the AES. Due to low item loadings with the previous version, the Customer Orientation subscale was not reduced and thus retained all of its items. Additionally, two items that were new on this version of the AES were added to the Customer Orientation subscale ("I should be able to choose how my knowledge is tested" and "I ought to be able to choose the courses required for my degree").

*Model 4: Reduced model*. The Customer Orientation subscale was reduced to four items, based on the loadings in Model 3. The other subscales retained the same reduced set of items as in Model 3.

*Model 5: Reduced model, new items added*. Two remaining items were new in this version of the AES. These items were added to the reduced model; "Even if I do not perform well, I should get a good grade if I worked hard" was added to Reward for Effort, and "I should have my instructor's cell phone number to contact him or her if I need help" was added to the Customer Service subscale.

*Model 6: Reduced model, with Cross-Loading*. Modification indices revealed that allowing item 8 ("If I do poorly in a course, the fault lies with my professor") to cross-load on both Responsibility Avoidance and Accommodation would improve fit.  However, this resulted

in the item having a relatively strong loading on Accommodation (.639) and a near-zero loading (-.008) on Responsibility Avoidance.

*Model 7: Reduced model, item 8 loading on Accommodation only*. Given the item's strong loading on Accommodation, we trimmed its path from Responsibility Avoidance.

*Model 8: Reduced model, removing item from Accommodation*. In order to have the same number of items on each subscale, we removed an item ("A professor should modify course requirements to help me achieve a better grade") from Accommodation. This was done on the basis of the item's relatively lower loading (.579) in comparison to the other items on that factor. This model was also tested in the second subsample and total sample.

*Model 9: Reduced model with General AE as correlated factor*. We added the General AE factor, with its six unique items, as a correlated factor. The specific factors retained the same specification as in Model 8. This model was also tested in the second subsample and total sample.

*Model 10: Bifactor model*. To better understand the General factor, we modeled a bifactor model, such that the General factor had its own unique items loading onto it, in addition to the rest of the items. The specific factors were modeled according to the reduced model tested in Model 8, with four items loading on each specific factor. The General factor was kept orthogonal to the six specific factors. This model was also tested in the second subsample and total sample.
